# Full-spectral genome analysis of natural killer/T cell lymphoma highlights impacts of genome instability in driving its progression

**DOI:** 10.1186/s13073-024-01324-5

**Published:** 2024-04-02

**Authors:** Zegeng Chen, He Huang, Huangming Hong, Huageng Huang, Huawei Weng, Le Yu, Jian Xiao, Zhao Wang, Xiaojie Fang, Yuyi Yao, Jia-Xing Yue, Tongyu Lin

**Affiliations:** 1 State Key Laboratory of Oncology in South China, Collaborative Innovation Center for Cancer Medicine, Guangdong Key Laboratory of Nasopharyngeal Carcinoma Diagnosis and Therapy, Sun Yat-Sen University Cancer Center, Guangzhou, 510060 China; 2 Department of Medical Oncology, Sun Yat-Sen University Cancer Center, Guangzhou, 510060 China; 3 Department of Medical Oncology, Sichuan Clinical Research Center for Cancer, Sichuan Cancer Hospital and Institute, Sichuan Cancer Center, Affiliated Cancer Hospital of University of Electronic Science and Technology of China, Chengdu, 610041 China; 4 Department of Medical Oncology, The Sixth Affiliated Hospital of Sun Yat-Sen University, Guangzhou, 510655 China

**Keywords:** Natural killer/T cell lymphoma, Genomic alteration, Genome instability, Chromothripsis, Molecular subtypes

## Abstract

**Background:**

Natural killer/T cell lymphoma (NKTCL) is a clinically and genetically heterogeneous disease with poor prognosis. Genome sequencing and mutation characterization provides a powerful approach for patient stratification, treatment target discovery, and etiology identification. However, previous studies mostly concentrated on base-level mutations in primary NKTCL, whereas the large-scale genomic alterations in NKTCL and the mutational landscapes in relapsed/refractory NKTCL remain largely unexplored.

**Methods:**

Here, we assembled whole-genome sequencing and whole-exome sequencing data from 163 patients with primary or relapsed/refractory NKTCL and compared their somatic mutational landscapes at both nucleotide and structure levels.

**Results:**

Our study not only confirmed previously reported common NKTCL mutational targets like *STAT3*, *TP53*, and *DDX3X* but also unveiled several novel high-frequency mutational targets such as *PRDM9*, *DST*, and *RBMX*. In terms of the overall mutational landscape, we observed striking differences between primary and relapsed/refractory NKTCL patient groups, with the latter exhibits higher levels of tumor mutation burden, copy number variants (CNVs), and structural variants (SVs), indicating a strong signal of genomic instability. Complex structural rearrangements such as chromothripsis and focal amplification are also significantly enriched in relapsed/refractory NKTCL patients, exerting a substantial impact on prognosis. Accordingly, we devised a novel molecular subtyping system (i.e., C0–C4) with distinct prognosis by integrating potential driver mutations at both nucleotide and structural levels, which further provides an informative guidance for novel treatments that target these specific driver mutations and genome instability as a whole.

**Conclusions:**

The striking differences underlying the mutational landscapes between the primary and relapsed/refractory NKTCL patients highlight the importance of genomic instability in driving the progression of NKTCL. Our newly proposed molecular subtyping system is valuable in assisting patient stratification and novel treatment design towards a better prognosis in the age of precision medicine.

**Supplementary Information:**

The online version contains supplementary material available at 10.1186/s13073-024-01324-5.

## Background

Natural killer/T cell lymphoma (NKTCL) is a unique subtype of non-Hodgkin lymphoma with poor prognosis, which predominantly occurs in East Asia and Latin America [1]. The occurrence of NKTCL is associated with Epstein-Barr virus (EBV) infection, although the actual mechanism remains elusive [2]. Currently, the asparaginase-based chemotherapy is used as the first-line treatment for NKTCL, which helps to notably improve the survival of NKTCL patients [1, 3]. Yet approximately 40–50% of patients, especially those in advanced NKTCL stages, do not respond well to this treatment and frequently experience relapse after the first-line treatment [4, 5]. Patients with relapsed/refractory NKTCL had poor prognosis with a median overall survival (OS) less than 1 year [6]. So far, there is a lack of progress in the development of targeted therapy for NKTCL, which urges a need for better understanding of the molecular pathogenesis of NKTCL.

Recent applications of genome sequencing on NKTCL patients revealed recurrent mutational targets such as RNA helicase (e.g., *DDX3X*), tumor suppressors (e.g., *TP53*), and genes involved in the JAK-STAT and RAS-MAPK signaling pathways, as well as epigenetic modulators (e.g., *KMT2C* and *KMT2D*) [7–9]. Among them, mutations in *DDX3X* were identified in ~ 20% NKTCL patients, and it has been shown that *DDX3X* mutations can result in cell-cycle progression and transcriptional activation of the NF-κB pathways [7]. Meanwhile, mutations in the JAK-STAT pathway were observed in ~ 30% of NKTCL patients, highlighting the importance of the JAK-STAT pathway underlines the development and progression of NKTCL [8, 10]. Interestingly, *TP53* was comparatively less mutated in NKTCL than other solid tumors, and those patients with *TP53* mutations are usually with advanced stages, which collectively suggests the *TP53* mutation as a secondary driver in NKTCL [11].

To date, most such sequencing-based NKTCL studies concentrated on primary NKTCL, whereas the mutational landscape of relapsed/refractory NKTCL remains largely unexplored. Therefore, a genome-wide survey and comparison of the relapsed/refractory against primary NKTCL is needed for uncovering main factors that influences the effectiveness of the current NKTCL treatment. Moreover, existing genomic studies on NKTCL majorly focused on single nucleotide variants (SNVs) and small insertion/deletions (INDELs), so it remains to be examined the prevalence and importance of other alterations such as copy number variants (CNVs) and structural variants (SVs) in NKTCL. The functional impacts of these genomic alterations can easily surpass those of SNVs and INDELs given their much larger genomic scales [12]. For example, SVs such as segmental deletion, insertion, and duplication can lead to high-level loss of tumor suppressors and amplification of oncogenes, therefore driving cancer development and metastasis [13]. The chromosomal gain/loss caused by aneuploidy can substantially disrupt the dosage balance of the cell and induces immediate mitotic stress and genome instability [14]. More dramatically, massive chromosomal rearrangements known as chromothripsis can rampantly disrupt gene integrity and epigenetic contexts, representing a mutational meltdown of cancer genomes [15]. Recently, the prevalence of extracellular circular DNA (eccDNA) in tumor cells has also caught attention, which represents a new forms of genome instability [16]. Multiple studies further demonstrated the occurrence of chromothripsis and eccDNA can contribute to treatment resistance and poor prognosis [17, 18]. So far, it is unclear if these dramatic genomic alterations are involved in the development of NKTCL.

In this study, we assembled the largest panel of NKTCL patients with either whole-genome sequencing (WGS) or whole-exome sequencing (WES) data available. Both primary and relapsed/refractory patients are included with well-documented clinical metadata. Altogether, this setup enabled us to systematically delineate and compare their genomic alteration landscapes of full spectrum (i.e., SNV, INDEL, CNV, SV), also including those dramatic genomic alterations such as aneuploidy, chromothripsis, and eccDNA. Finally, a novel molecular subtyping system was devised by integrating driver mutations at both nucleotide and structure levels. Our genomic characterization and molecular subtyping collectively highlight the need of introducing novel treatments such as immune checkpoint inhibitor and other immune-combined therapies that exploit the high mutation burden and genome instability of relapsed/refractory NKTCL.

## Methods

### Patients and NGS

Patients diagnosed with NKTCL during 2010–2020 at Sun Yat-sen University Cancer Center, Sichuan Cancer Hospital, and the Sixth Affiliated Hospital of Sun Yat-sen University were retrospectively enrolled in this study. A total of 42 patients (19 primary, 23 relapsed/refractory) were enrolled and subjected to WES. Technical details of DNA extraction and NGS are described in the Additional file 1: Supplementary materials and methods. In addition, raw NKTCL WGS and WES reads from several published studies were retrieved and reanalyzed as well, which include (1) the International Cancer Genome Consortium (ICGC), with 23 WGS datasets (20 primary, 3 relapsed/refractory) [19]; (2) the European Genome-phenome Archive (EGA), with 12 WGS datasets (2 primary, 10 relapsed/refractory) [20]; and (3) the National Omics Data Encyclopedia (NODE), which includes data on primary patients with 36 WGS and 50 WES datasets [8]. The clinical data associated with these studies were also retrieved. The sequencing method for each sample enrolled, along with the corresponding average sequencing depth, is presented in Additional file 2: Table S1. In total, 71 patients (primary: *n* = 58, relapsed/refractory: *n* = 13) with WGS data (mean sequencing depth: 52 ×) and another 92 patients (primary: *n* = 69, relapsed/refractory: *n* = 23) with WES (mean sequencing depth: 101 ×) data were included in our analysis (Additional file 2: Table S1).

### Reference genome and quality control

Careful quality control was applied during our data integration and processing. The reference genome, software, and parameters we used for quality control are presented in Additional file 1: Supplementary materials and methods. Minimal on-target mapping depth cutoffs of 20 × for WGS data and 30 × for WES data was further used to filter samples with insufficient sequencing coverage (if any).

### Somatic mutation calling

Somatic SNVs and INDELs were detected with MuTect2 (v4.1.0.0) [21] and annotated by ANNOVAR (v2020Jun08) [22]. MSIsensor-pro (v1.2.0) [23] was utilized to evaluate microsatellite instability (MSI), generating an MSI score for each sample. SigProfiler Tools (v1.2.14) [24] were used for extracted and visualized mutational signatures. Somatic CNVs were called by CNVkit (v0.9.9) [25] and ASCAT (v3.1.1) [26]. The KEGG pathway enrichment analysis for gene-level CNVs was performed by clusterProfiler (v4.7.1.3) [27]. The aneuploidy score was calculated using the get_Aneuploidy_score() function implemented in sigminer (v2.1.9) [28]. For WGS data, we also examined the landscape of SVs using manta (v1.6.0) [29], profiled chromothripsis events with ShatterSeek (v1.1) [15], and detected and classified focal amplifications with AmpliconArchitect (v1.3.r1) [30] and AmpliconClassifier (v0.4.13) [31]. The details of mutation calling are described in Additional file 1: Supplementary materials and methods.

### Survival and statistical analysis

Progression-free survival (PFS) was assessed from the date of diagnosis of NKTCL until first relapse, progression, or death from any cause. Overall survival (OS) was measured from the date of diagnosis to the date of death from any cause. PFS and OS were estimated using the Kaplan–Meier method and statistically compared using the log-rank test implemented in survival (v3.5–5; https://cran.r-project.org/web/packages/survival/index.html).

### Non-negative matrix factorization consensus clustering

To classify patients based on their mutational profiles, we adopted a modified non-negative matrix factorization (NMF) consensus clustering algorithm [32]. For each patient, its clustering membership was identified based on the calculated cophenetic coefficients for *k* = 2–9 clusters and silhouette values. Samples lacking driver mutations were defined as cluster C0. ComplexHeatmap (v2.14.0) [33] was used to visualize the clustering results.

## Results

### Higher nucleotide mutational load in relapsed/refractory NKTCL patients than the primary NKTCL patients

The clinical features of all 163 enrolled patients were examined and a side-by-side comparison among different cohorts was performed. Majority of patients (97.1%, 136 out of 140 patients whose treatment information is accessible) received first-line asparaginase-based chemotherapy. The NODE and ICGC cohorts are mostly comprised of primary patients, whereas the NKT_project and EGA cohorts contain more relapsed/refractory patients (Additional file 2: Table S2). Their prognoses vary among different cohorts (Additional file 3: Fig. S1). To evaluate the impacts of different clinical features on the overall survival, we performed both univariate and multivariate Cox regression analyses. While the analysis on different disease stages, primary vs. relapsed/refractory status, prognostic scores, and patient cohorts all appear as significant in the univariate analysis, only the relapsed/refractory status is significantly associated with poor prognosis in the multivariate analysis (Additional file 2: Table S3). Patients with relapsed/refractory NKTCL generally show more advanced Ann-Arbor stage and higher IPI and PINK scores compared with their primary counterparts (Additional file 2: Table S4). The overall survival (OS) of relapsed/refractory patients is significantly worse in comparison (5-year OS rate 57.9% versus 27.4%, *P* = 0.003, Additional file 3: Fig. S1).

**Fig. 1:**
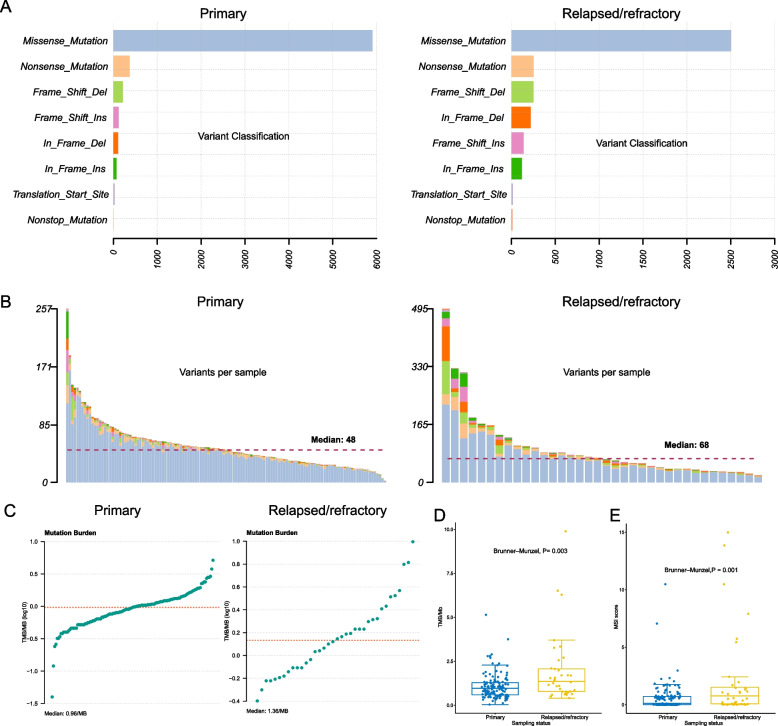
Characterization of somatic mutations and tumor mutation burden in NKTCL. **A** The per-sample numbers of different categories of non-synonymous somatic mutations in primary (left) and relapsed/refractory (right) NKTCL. **B** Per-sample distribution of different categories of non-synonymous mutations in patients with primary (left) and relapsed/refractory (right) NKTCL. The dashed line denotes the median value. **C** The tumor mutation burden (TMB) profiles of primary (left) and relapsed/refractory (right) patients. The dashed line denotes the median value. **D** The comparison of TMB levels between the primary and the relapsed/refractory group. Brunner–Munzel test was applied for assessing the statistical significance. **E** The comparison of MSI score between the primary and the relapsed/refractory group. Brunner–Munzel test was applied for assessing the statistical significance

For primary patients (*n* = 127), a total of 6826 nonsilent somatic mutations were identified, including 5904 missense mutations, 373 nonsense mutations, 340 out-frame INDELs, and 177 in-frame INDELs. For relapsed/refractory patients (*n* = 36), we identified a total of 3512 nonsilent mutations, comprising 2503 missense mutations, 254 nonsense mutations, 392 out-of-frame INDELs, and 366 in-frame INDELs. The per-sample mutation count in relapsed/refractory patients is significantly higher than that in the primary patients (per-sample median: 68 vs. 48, Brunner–Munzel test, *P* = 0.003, Fig. 1A, B). Likewise, a higher tumor mutation burden (TMB) level was also found in relapsed/refractory patients (Brunner–Munzel test, *P* = 0.003, Fig. 1C, D). To account for the potential confounding factor introduced by sequencing method difference (i.e., WES vs. WGS), we conducted separate analyses for patients with WES and WGS data. For patients with WES data, relapse/refractory patients show a median count of 55 nonsilent somatic mutations, compared to 40 in the primary group (Additional file 3: Fig. S2). Additionally, the relapse/refractory patients exhibit significantly higher levels of TMB than the primary group (per-sample median: 1.1 vs. 0.8 per megabase, Brunner–Munzel test, *P* = 0.022, Additional file 3: Fig. S2). For patients with WGS data, we also observed a higher median count of nonsilent somatic mutations in the relapse/refractory group (per-sample median: 103 vs. 60, Additional file 3: Fig. S2) compared with the primary group. Significant disparity also exists at the TMB level (per-sample median: 2.1 vs. 1.2 per megabase, Brunner–Munzel test, *P* = 0.017, Additional file 3: Fig. S2). Taken together, these results suggest a higher nucleotide-level mutational load in relapsed/refractory NKTCL patients. In addition, we also noticed that the relapsed/refractory patients exhibit significantly higher MSI scores compared to their primary counterparts (per-sample median: 0.755 vs. 0.120, Brunner–Munzel test, *P* = 0.001, Fig. 1E). Such differences remained as significant when considering patients with WES and WGS data separately (Additional file 3: Fig. S2). This observation further highlights the higher levels of genome instability in the relapsed/refractory NKTCL patients compared with their primary counterparts.

### Enrichment of mutational signatures associated with DNA damage repair defection in relapsed/refractory NKTCL patients

**Fig. 2:**
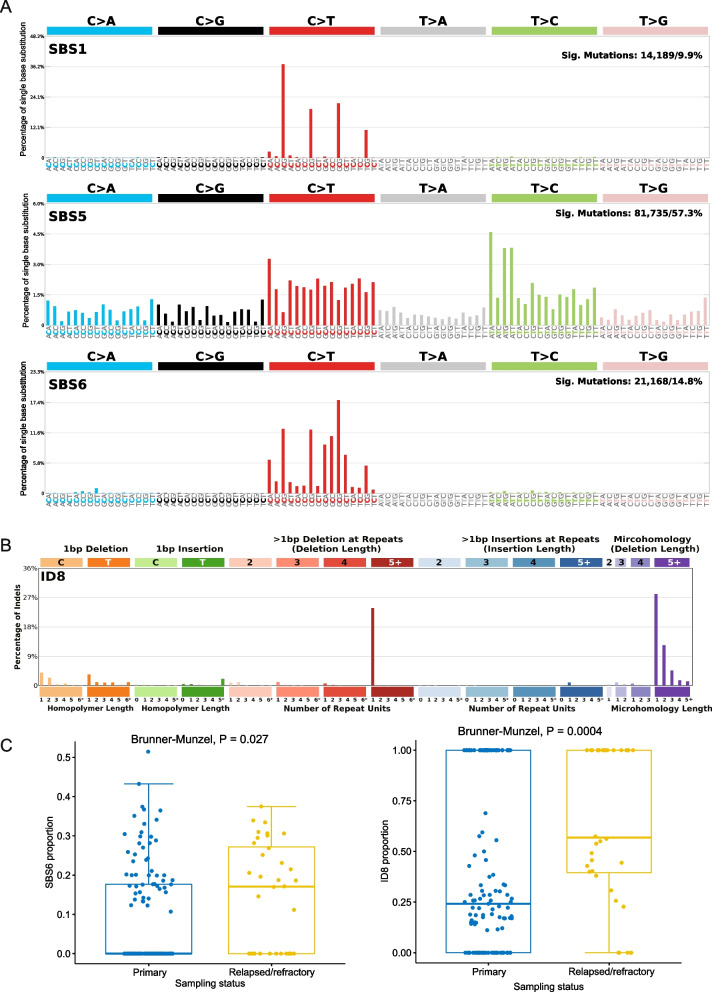
Mutational signatures operating in NKTCL. **A** Major SBS signatures (SBS1, 5, 6) identified from the 163 NKTCL patients. **B** Major INDEL signature (ID8) identified from the 163 NKTCL patients. **C** Proportions of mutations bearing the SBS6 and ID8 signatures in the primary and relapsed/refractory groups respectively. Brunner–Munzel test was applied for assessing the statistical significance

We next examined the spectrum and context of SNVs. We saw a strong signal of C- > T transition that was also observed in other solid tumors [34], suggesting possible involvement of epigenetic alterations like DNA methylation in the pathogenesis of NKTCL. Three prominent single base substitution (SBS) signatures popped up among all samples: SBS1, SBS5, and SBS6, all of which directly contribute to C- > T mutations (Fig. 2A). Etiologically, SBS1 is explained by spontaneous or enzymatic deamination of 5-methylcytosine (m5C) to thymine [35]. SBS5 often correlates with SBS1, with potential link to tobacco smoking [36]. SBS6 is frequently observed in microsatellite unstable tumors with association to DNA repair defection [36]. We also analyzed mutational signatures of INDELs and identified the ID8 signature as the most dominant signature (Fig. 2B). ID8 is related to the repair of double strand breaks (DSBs) by non-homologous DNA end-joining (NHEJ) and bears similarity to radiation induced mutations [36, 37]. Notably, both SBS6 and ID8 are especially enriched in patients with relapsed/refractory NKTCL (Fig. 2C). Given that these two signatures are both associated with defective DNA damage repair, this observation hints that genes responsible for DNA damage repair might be a common mutational targets in relapsed/refractory NKTCL patients, which also explains the higher mutational load observed in these patients.

### Shared and distinct coding mutations between primary and relapsed/refractory NKTCL

**Fig. 3:**
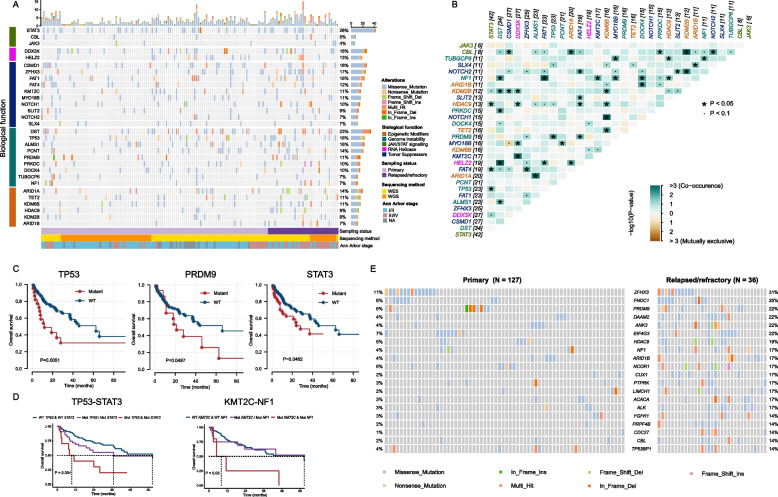
Mutational landscape of primary and relapsed/refractory NKTCL. **A** The top panel shows the frequency of non-synonymous mutations in each sample. The main panel shows the occurrence of major somatic mutations across different samples, with different colors correspond to different mutation categories. Genes involved in these mutations are grouped based on their biological functions indicated on the left. The overall frequency of these somatic mutations across all examined samples are shown on the right. The lower panel indicates the sampling status (primary vs. relapsed/refractory), sequencing methods (WGS vs. WES), and Ann Arbor stage (I/II vs. III/IV) of each sample. **B** The co-occurrence and mutual exclusivity patterns among the frequent mutations. The font colors of gene names represent the biological function depicted in (**A**). **C** Kaplan–Meier curves of overall survival (OS) by the mutation status of *TP53*, *PRDM9*, and *STAT3*. **D** Kaplan–Meier curves of OS by the mutation status of TP53-STAT3 and KMT2C-NF1. **E** The mutational landscape of somatic mutations with significant higher frequency in relapsed/refractory patients than in primary patients (Fisher’s exact test, *P* < 0.05). The mutational frequency of these genes in primary patients is shown on the left, while their corresponding mutational frequencies in relapsed/refractory patients are shown on the right. The mutated genes are ranked by the mutational frequency in the latter group

Regarding common mutated gene targets in NKTCL, we compiled a short list of recurrent mutated genes by filtering out those genes mutated in < 5 patients or frequently appeared in non-disease-specific WES datasets [38] (Fig. 3A and Additional file 3: Fig. S3). Consistent with previous reports [39], *STAT3* comes up as the most frequent mutated gene (28%) with most mutations occurred in its SH2 domain (47 of 53 mutations) (Additional file 3: Fig. S3B). Other genes of the JAK-STAT signaling pathway (e.g., *CBL* and *JAK3*) are also frequently mutated. We detected *TP53* mutations in 16% of patients, with the mutated sites predominantly located in its P53 DNA-binding domain (Additional file 3: Fig. S3C). Of the helicase gene family, we recaptured the RNA helicase gene *DDX3X* (18%) as a recurrent mutation target, with various missense mutations affecting its ATP-binding helicase domain (Additional file 3: Fig. S3D). Meanwhile, mutations in another helicase gene *HELZ2* are also detected in 13% of patients. Other identified recurrent mutated genes include the following: tumor suppressors (*CSMD1*, 18%; *ZFHX3*, 17%; *FAT1*, 16%; *FAT4*, 12%; *KMT2C*, 11%; *MYO18B*, 11%; *NOTCH1*, 10%; *SLIT2*, 9%; *NOTCH2*, 7%; *SLX4*, 7%), genome instability modulators (*DST*, 23%; *ALMS1*, 16%; *PCNT*, 14%; *PRDM9*, 11%; *PRKDC*, 10%; *DOCK4*, 10%; *TUBGCP6*, 7%; *NF1*, 7%), and epigenetic modifiers (*ARID1A*, 14%; *TET2*, 11%; *KDM6B*, 11%; *HDAC9*, 9%; *KDM2B*, 8%; *ARID1B*, 7%).

We further investigated the co-occurrence and mutual exclusivity among these common mutations in NKTCL. We found 44 gene pairs showing significant co-occurrence mutations (Fisher’s exact test, *P* < 0.05), with *NOTCH1*-*KDM6B* (*P* = 0.001), *CBL*-*KDM2B* (*P* = 0.001), and *DST*-*HELZ2* (*P* = 0.001) being on the top of the list (Fig. 3B). A previous study highlighted *KDM6B*’s crucial role in supporting *NOTCH1*-driven T cell acute lymphoblastic leukemia [40]. The synergistic effect of *NOTCH1* and *KDM6B* mutations therefore may accelerate lymphoma progression by activating oncogenic pathways via chromatin remodeling. Other interactions also likely exacerbate oncogenic stress by fostering lymphoma cell proliferation. Future studies are needed to evaluate the therapeutic potential of targeting these interactions. To explore the prognostic value of these recurrent mutations and their co-occurring combinations, we applied the Kaplan–Meier analysis and discovered that mutations in *TP53*, *PRDM9*, and *STAT3* are significantly associated with worse OS (Fig. 3C). While such association has been previously reported for *TP53* and *STAT3* [39, 41], our study provides the first evidence for the prognosis value of *PRDM9* aberration. We found patients with *STAT3*-*TP53* co-mutations exhibited a worse prognosis than patients with a single or no mutation of *STAT3* and *TP53* (*P* = 0.004) (Fig. 3D). Similarly, patients carrying *KMT2C*-*NF1* co-mutations are significantly associated with poor prognosis compared to those carrying either a single mutation or no mutation in these two genes (*P* = 0.03) (Fig. 3D).

We found 68 genes that are significantly more likely to be mutated in relapsed/refractory patients (Fisher’s exact test), which include genome instability modulators (*CDC27*, *P* = 0.002; *PRPF4B*, *P* = 0.006; *PRDM9*, *P* = 0.009; *NF1*, *P* = 0.015) and epigenetic modifiers (*ARID1B*, *P* = 0.015; *HDAC9*, *P* = 0.009; *NCOR1*, *P* = 0.040) as well as tumor suppressors (*CUX1*, *P* = 0.001; *ZFHX3*, *P* = 0.007; *PTPRK*, *P* = 0.008; *CBL*, *P* = 0.013; *TP53BP1*, *P* = 0.043) (Fig. 3E). Given that relapsed/refractory patients are generally associated with more advanced Ann Arbor stages (Fisher’s exact test, *P* < 0.001), it is likely that these differentially mutated genes play important roles in promoting the progression of NKTCL. We also examined the co-occurrence and mutual exclusivity among these 68 genes, which reveals a series of co-occurrence mutations (Fisher’s exact test, *P* < 0.05, Additional file 3: Fig. S3E) such as *ZFHX3*-*FNDC1* (*P* < 0.001), *HDAC9*-*FGFR1* (*P* = 0.002), and *ACACA*-*NF1* (*P* = 0.02). The prevalence of these co-occurring mutations underscores the role of synergistic mutations in driving the progression and evolution of NKTCL.

In addition, the infection status of Epstein–Barr virus (EBV) has also been reported to be significantly associated with the prognosis of NKTCL [42]. For the patients with accessible EBV-infection data (*n* = 115), we evaluated the correlation between patients’ peripheral blood EBV-infection status and their NKTCL mutational profiles. For those most-frequently mutated genes in NTKCL such as *STAT3*, *DST*, *CSMD1*, *DDX3X*, no significant difference can be detected regarding their mutational frequency between the EBV-positive and EBV-negative groups. However, specific gene mutations are notably enriched in the EBV-positive group (Fisher’s exact test), including *UBR4* (*P* = 0.010), *C2orf16* (*P* = 0.019), *CACNA1E* (*P* = 0.036), *UTP20* (*P* = 0.014), and *COL6A5* (*P* = 0.030). Conversely, certain genes such as *HERC2* (*P* = 0.047) and *RRP1* (*P* = 0.018) are significantly enriched in the EBV negative group (Additional file 3: Fig. S3F). *UBR4* has been reported to interact with viral proteins that are important for viral life cycle [43]. In EBV-positive NKTCL, frequent mutations in *UBR4* could influence the viral manipulation of host cellular mechanisms, potentially leading to oncogenic processes such as immune evasion and unregulated cellular proliferation. The positive association between *UBR4* mutations and EBV infection underscores the role of *UBR4* in the pathogenesis of EBV-associated malignancies and highlights its potential value for therapeutic intervention.

### Higher copy number alteration and aneuploidy occurrences in relapsed/refractory patients

**Fig. 4:**
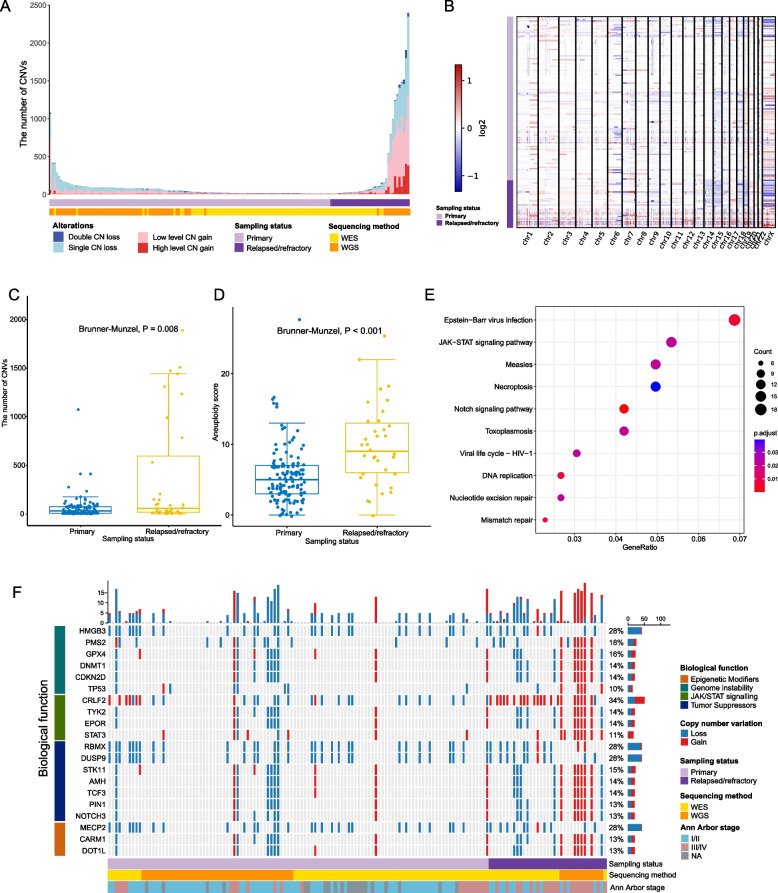
The landscape of copy number variation in NKTCL. **A** Distribution of different categories of CNVs across the 163 NKTCL patients. The CNV categories employed here include single CN loss (CN = 1 copy), double CN loss (CN = 0), low-level CN gain (CN = 3), and high-level CN gain (CN > 3 copies). **B** Heatmap of CNVs in patients with primary and relapsed/refractory NKTCL. **C** The per-sample CNV number comparison between the primary and the relapsed/refractory groups. Brunner–Munzel test was applied for assessing the statistical significance. **D** The per-sample aneuploidy score comparison between the primary and the relapsed/refractory groups. Brunner–Munzel test was applied for assessing the statistical significance. **E** KEGG pathway enrichment analysis of gene-level CNVs for patients with NKTCL. **F** Gene-level CNV landscape across the 163 NKTCL patients

We profiled the somatic CNVs landscape based on both WGS and WES data. The number of CNVs vary substantially across different samples, with an average of 138.2 CNVs identified per sample (Fig. 4A). We classified these CNVs into four categories: single CN loss (CN = 1 copy), double CN loss (CN = 0), low-level CN gain (CN = 3), and high-level CN gain (CN > 3 copies). Nearly 90% of CNVs are single CN loss (42.5%) and low-level CN gain (44.8%), which is different from previous observations in other solid tumors where ~ 52.4% cases were detected with amplicons of CN ≥ 5 [44]. We found a significantly higher CNV burden in relapsed/refractory patients (per-sample median: 58.5 vs. 33, Brunner–Munzel test, *P* = 0.008, Fig. 4B, C), reflecting their elevated genome instability. Consistent results were obtained when analyzing the WGS and WES datasets separately (Additional file 3: Fig. S4). Aneuploidy is an extreme type of CNV that spans over entire chromosome arms with substantial impact on genome instability. We observed a significantly higher aneuploidy level for relapsed/refractory patients (median score: 9) than their primary counterparts (median score: 5) (Brunner–Munzel test, *P* < 0.001, Fig. 4D).

At the gene-level, we identified several genes with recurrent CN changes. These genes are associated with biological function such as genome integrity (*HMGB3*, *PMS2*, *GPX4*, *DNMT1*, *CDKN2D*, and *TP53*), JAK-STAT signaling (*CRLF2*, *TYK2*, *EPOR*, *STAT3*), tumor suppressors (*RBMX*, *DUSP9*, *STK11*, *AMH*, *TCF3*, *PIN1*, *NOTCH3*), and epigenetic modifiers (*MECP2*, *CARM1*, *DOT1L*). At the pathway-level, these genes are significantly enriched in pathways involved in EBV infection, JAK-STAT signaling, and DNA replication and mismatch repair (Fig. 4E). When comparing the CNV profiles between primary and relapsed/refractory patients, we found genes such as *CRLF2*, *PMS2*, and *STK11* show significantly higher propensity for copy number alterations in relapsed/refractory patients (Fig. 4F). Among these genes, the frequency of *CRLF2* CN gain in relapsed/refractory patients is significantly higher than that in the primary group (21/36 vs. 8/127, *P* < 0.001). *CRLF2* overexpression has been widely confirmed to be associated with adverse outcomes in B cell acute lymphoblastic leukemia (B-ALL) [45]. It has been shown that high-dose of thymic stromal lymphopoietin (TSLP) can induce apoptosis, impeding the proliferation and migration of CRLF2 B-ALL tumor cells [46]. Furthermore, the synergistic combination of the JAK1/2 kinase inhibitor ruxolitinib with conventional treatment has demonstrated enhanced efficacy in CRLF2-altered B-ALL [47]. These findings offer valuable insights into potential treatment options for patients with relapsed/refractory NKTCL.

By leveraging the exact copy number, segment size, and heterozygosity status of these CNVs [48], we summarized the CN signatures for each NKTCL sample (Additional file 3: Fig. S5A). We found CN1, CN2, CN5, and CN9 are among the most prominent signatures. Notably, CN2 (tetraploid genome, i.e., whole genome doubling), CN5 (chromothripsis), and CN9 (chromosomal instability in diploid genome) are all associated with genome instability [49]. In particular, CN9 is significantly more predominant in patients with relapsed/refractory NKTCL (Brunner–Munzel test, *P* = 0.041; Additional file 3: Fig. S5B). Patients with the CN9 signature show significantly inferior OS (Additional file 3: Fig. S5C).

### Landscape of canonical and complex structural rearrangements in NKTCL patients

**Fig. 5:**
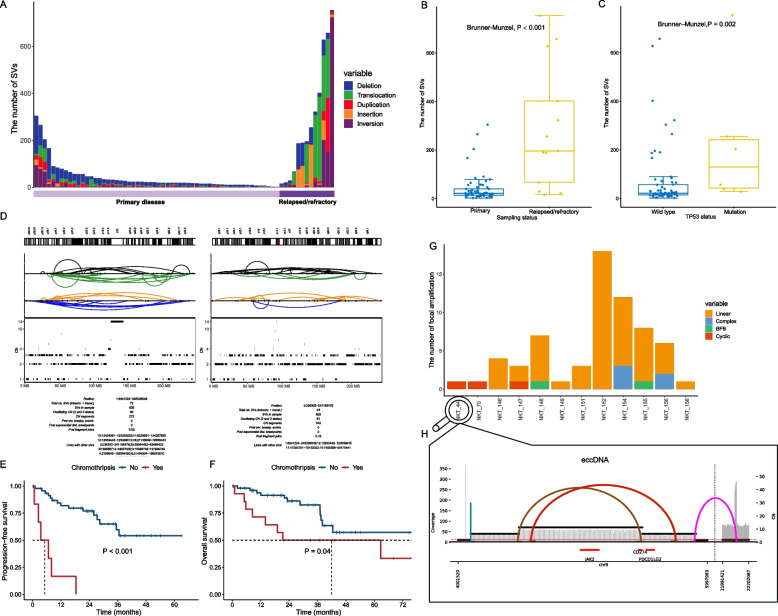
The landscape of canonical and complex structure variation in NKTCL. **A** Distribution of canonical structural variants (SVs) across the 71 NKTCL patients with WGS data. The canonical SV types employed here include insertion, deletion, duplication, inversion, and translocation. **B** The comparison of per-sample canonical SV numbers between the primary and relapsed/refractory patient groups. Brunner–Munzel test was applied for assessing the statistical significance. **C** The comparison of per-sample canonical SV numbers between the wildtype and mutated *TP53* patient groups. Brunner–Munzel test was applied for assessing the statistical significance. **D** An exemplary chromothripsis event detected in one NKTCL patient. The chromosome ideograms of the affected regions (on chromosomes 1 and 2) are shown at the top. Different subtypes of SVs are represented by connected lines illustrated in the middle: black, head-to-head inversion (+ / +); green, tail-to-tail inversion (− / −); orange, deletion-like (+ / −); blue, duplication-like (− / +). The copy number profile of the corresponding regions is depicted at the bottom, where the horizontal and vertical axes represent the chromosome coordinates and copy number respectively, while each CN segment is represented by a black bar. Detail information for the depicted region is presented as a table at the bottom. **E** Kaplan–Meier curves of progression-free survival (PFS) by the attribute of chromothripsis in the NKTCL patients with WGS data. **F** Kaplan–Meier curves of overall survival (OS) by the attribute of chromothripsis in the NKTCL patients with WGS data. **G** Detailed characterization of 12 samples identified with focal amplifications, for which focal amplicons were further classified into linear, complex, cyclic (eccDNA), and breakage fusion bridge (BFB). **H** The genome browser track derived from one patient (denoted as NKT_44) depicted by AmpliconArchitect. The connected lines indicate potential structure combinations in eccDNA amplicons. The horizontal axis represents chromosome coordinates. The left vertical axis represents the depth of coverage, while the right vertical axis denotes the copy number

For NKTCL patients whose WGS data is available, we next extend our analysis to canonical SVs such as insertions, deletions, duplications, inversions, and translocations. Altogether, we identified a total of 6131 events across all 71 NKTCL patients (mean: 86.3 per patient) with 29% of them being segmental deletions (Fig. 5A). Consistent with our observations based on SNV, INDELs, and CNVs, we found the overall SV burden of relapsed/refractory patients is significantly higher than that of primary patients (Brunner–Munzel test, *P* < 0.001, Fig. 5B). Also, patients with *TP53* mutations show more SVs than those without *TP53* mutations (Brunner–Munzel test, *P* = 0.002), highlighting the impact of *TP53* inactivation on SV accumulation (Fig. 5C).

SVs can promote tumorigenesis and progression by forming fused oncogenes or by disrupting tumor suppressor genes [50]. Indeed, we identified a set of cancer-related genes recurrently interrupted by SV breakpoints (Additional file 2: Table S5). For example, *CD274* (PD-L1) encodes an immune inhibitory receptor ligand, and its expression is associated with tumor immune escape in many types of cancers [51]. We identified SVs in this gene in 17.2% of primary and 7.7% of relapsed/refractory patients respectively, with 38.4% of such SV cases being tandem duplications. *LRP1B* disruption has been shown as a potential contributor to the emergence of chemotherapy resistance in ovarian cancer [52]. Our findings reveal that *LRP1B* is also frequently interrupted by SV breakpoints in NKTCL, notably to a much larger extent in relapsed/refractory patients (6.9% in primary versus 30.8% in relapsed/refractory; Fisher’s exact test, *P* = 0.034). Therefore, the higher SV burden of *LRP1B* in NKTCL patients, especially those relapsed/refractory ones, likely serves to reduce chemotherapy sensitivity and therefore to lead to treatment failures. In addition, we found *RBFOX1* translocations also occur much more frequently in relapsed/refractory NKTCL patients (1.7% in the primary vs. 30.8% in relapsed/refractory; Fisher’s exact test, *P* = 0.003). *RBFOX1* promotes mRNA stability and it is down-regulated across multiple cancers types [53]. The frequent translocation observed in this gene in NKTCL is consistent with such pan-cancer pattern. This highlights the importance and potential therapeutic value for restoring post-transcriptional homeostasis in anticancer treatment.

We further extended our analysis to more complex SV types such as chromothripsis and eccDNA. We identified chromothripsis in 15 out of 71 patients (21.1%), with a significantly higher identification rate in the relapsed/refractory group (primary: 8/58 vs. relapsed/refractory: 7/13; Fisher’s exact test, *P* = 0.004). Chromosomes 1, 2, and 7 are frequently impacted by chromothripsis (Fig. 5D). *TP53* mutation has been reported to be associated with chromothripsis in solid tumors [54]. Concordantly, we found 50% (3/6) of *TP53*-mutated NKTCL patients have chromothripsis, whereas only 18.5% (12/65) of those NKTCL patients with wild-type *TP53* have chromothripsis, underscoring the risk of *TP53* mutation in triggering nuclear catastrophe. Patients suffer from chromothripsis show significantly inferior PFS and OS, highlighting the role of chromothripsis in promoting NKTCL progression (Fig. 5E, F).

Recent studies reported that chromothripsis could serve as a precursor for focal amplifications, which further promote the rapid amplification of oncogenes [55]. We detected a total of 65 focal amplification events in 12 out of 71 NKTCL patients, with 7 of them also have chromothripsis. Such focal amplifications are much more common in relapsed/refractory patients (9/13) than in primary patients (3/58) (69.2% vs 5.2%, *P* < 0.001, Fisher’s exact test). The detailed count and subtype of focal amplifications are heterogeneous across different patients, with linear focal amplifications being the most dominant subtype (*n* = 55) (Fig. 5G). As a special subtype of focal amplifications, eccDNA has been reported to be an important oncogenic driver that also contributes to drug resistance [18, 56]. We found eccDNA in 3 out of 12 patients with focal amplifications. Remarkably, in one patient (denoted as NKT_44) with eccDNA, the *JAK2*, *CD274* (PD-L1), and *PDCD1LG2* (PD-L2) genes all got substantially amplified with eccDNA (Fig. 5H). JAK2 is a critical component of the JAK-STAT signaling pathway whose over-activation can lead to NKTCL, while the amplification of *CD274* (PD-L1) and *PDCD1LG2* (PD-L2) genes will foster immune escape. Therefore, for this particular case, both JAK-STAT over-activation and immune escape contribute to the pathogenesis of NKTCL.

### Integration of genomic alterations of full spectrum to derive a novel molecular subtyping system for NKTCL

**Fig. 6:**
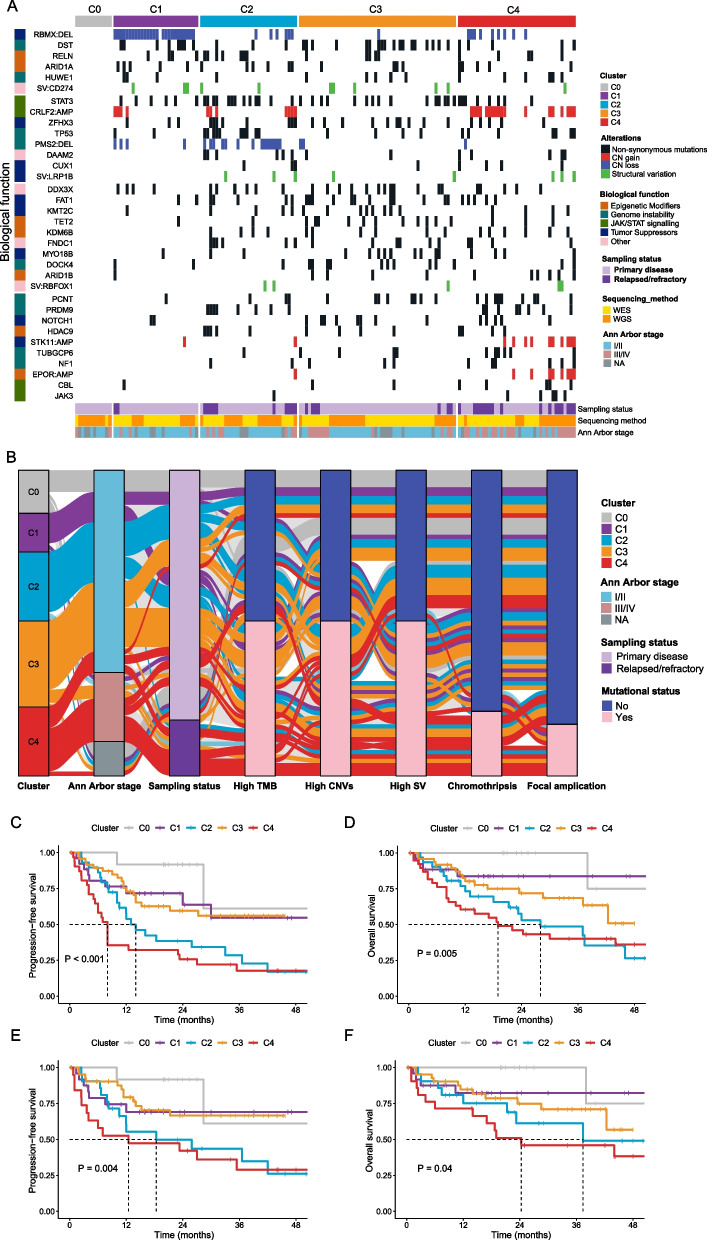
Identification of clusters of NKTCL with coordinate genomic alterations. **A** Nonnegative matrix factorization clustering was carried out using somatic SNVs, INDELs, CNVs, and SVs in the 163 NKTCL patients (columns). Samples without candidate alterations were defined as cluster C0. Clusters C1–C4 with their associated representative genetic alterations are visualized. **B** Sankey-diagram of the clinical and mutational characteristics for NKTCL patients. The eight columns from left to right represent molecular subtyping clusters, Ann Arbor stage, sampling status, TMB, CNV, SV, chromothripsis, and focal amplifications respectively, with the total height represents the full 71 WGS samples. The curves with different colors show the correspondence relationship among different characteristics and molecular subtyping clusters. **C** Kaplan–Meier curve of PFS and **D** OS of all NKTCL patients from different molecular subtyping clusters. **E** Kaplan–Meier curve of PFS and **F** OS of primary NKTCL patients from different molecular subtyping clusters

Given that we have systematically characterized genomic alteration landscapes of full spectrum (SNVs, INDELs, CNVs, and SVs) for primary and relapsed/refractory NKTCL patients, we further evaluated their prognostic values with NMF clustering [32]. A total of 151 patients were successfully classified into four clusters (C1–C4) (Fig. 6A). As for the remaining 12 patients, no characteristic driver genetic alterations were identified and therefore we classified them as the C0 cluster, although their genomes did show substantial alterations (mean SNVs = 26.4 per sample, mean CNVs = 63.6 per sample, mean SVs = 9.6 per sample) as those of other patients. All patients classified as C0 are primary patients. The C1 cluster is characterized by the mutations in *DST*, *RELN*, *ARID1A*, and CN loss of *RBMX*. The *CD274* (PD-L1) SVs are also highly noticeable in this cluster. The C2 cluster is represented by the mutations involving the JAK-STAT pathway, including *STAT3* and CN gain of *CRLF2*. The C3 cluster is associated with mutations in epigenetic modifiers such as *TET2*, *KDM6B*, and *ARID1B* as well as the mutations of tumor suppressors including *FAT1* and *KMT2C*. Finally, the C4 cluster is characterized by the CN gain of *STK11* and *EPOR* and mutations in genomic instability including *PCNT*, *PRDM9*, *TUBGCP6*, and *NF1*. Interestingly, patients from the C4 cluster predominantly have advanced-staged NKTCL with many of them being relapsed/refractory, whereas most patients from C1–C3 clusters have primary NKTCL (Fig. 6A). In accordance to this observation, the C4 cluster is also associated with the worst prognosis and higher PINK and IPI scores (Additional file 3: Fig. S6). Taken together, we found relapsed/refractory NKTCL patients are characterized by high levels of TMB, CNVs, and SVs, as well as higher propensity for chromothripsis and focal amplifications, reflecting their severe genome instability (Fig. 6B).

With this molecular subtyping analysis, we mainly want to identify characteristic genomic alterations that helps to classify patients and potentially guide their treatment strategies, which is the ultimate goal of precision medicine. Nevertheless, it is also interesting to test if our molecular subtyping system captures the inherent factors that determines patients’ prognosis. First, we found patients from these five clusters (i.e., C0–C4) show clear differences in PFS (*P* < 0.001, Fig. 6C) and OS (*P* = 0.005, Fig. 6D). Moreover, by excluding relapsed/refractory patients and performing the survival analysis for the 127 primary patients only, we found our molecular subtyping systems is still capable of differentiating the prognostic performance of primary NKTCL patients in terms of both PFS (*P* = 0.004, Fig. 6E) and OS (*P* = 0.04, Fig. 6F). These results underscore the value of our molecular subtyping system for future clinical practice on NKTCL.

## Discussion

In this study, we comprehensively characterized the first full-spectral NKTCL somatic mutational landscape for NKTCL. Not only did we validate the previously reported common NKTCL mutational targets such as *STAT3*, *TP53*, and *DDX3X* [7, 8, 39], we also identified a list of novel mutational targets and mutational types across both nucleotide and structure levels. Moreover, it is also the first systematic genomic comparison between primary and relapsed/refractory NKTCL patients. Notably, we found significant differences in both overall mutation burden and specific mutational signatures between the primary and relapsed/refractory NKTCL patients, with the latter group exhibiting strong signals of genome instability. Complex structural rearrangements like chromothripsis and focal amplification are predominantly enriched in relapsed/refractory NKTCL patients. Our results echo with findings of a recent pan-cancer study, which also found signs of genome integrity loss in various refractory cancers [57]. Therefore, a significant proportion of relapsed/refractory patients are likely not responding well to traditional treatments. Instead, immune checkpoint therapy will provide a promising alternative solution. In support of this idea, recent studies that applied anti-PD-1/PD-L1 treatments to relapsed/refractory NKTCL patients have observed marked survival improvements [58, 59]. Regretfully, these studies are mostly retrospective ones either with limited samples or being single-arm clinical trials only, and the objective response rates are still unsatisfactory. Large-scale prospective studies are needed in future to explore and evaluate more effective combination treatment.

Several emerging molecules that are currently being developed to target biological pathways implicated in genomic instability. A subset of these molecules have advanced to the clinical trial stage, including PARP, CDK, and Aurora kinase inhibitors [60–62]. Among them, PARP inhibitors have demonstrated promising efficacy for ovarian, prostate, and breast cancers with homologous recombination deficiency (HRD) [60, 63, 64], suggesting the value of targeting DNA repair deficiency for antitumor therapy. Likewise, several highly selective CDK4/6 inhibitors have also been approved for the treatments in metastatic breast cancer [65]. In addition to these targeted therapies, several clinical trials recently demonstrated the promising efficacy of the combination of immunotherapy along with chemotherapy drugs targeting genome instability for cancer treatments [66, 67]. Given that multiple genes responsible for cell cycle control and DNA damage repair are recurrently mutated in NKTCL, a joint treatment strategy of combining genomic instability targeted drugs and immune checkpoint inhibitors may provide better efficacy when treating relapsed/refractory NKTCL.

In this study, we also identified several co-mutation gene pairs with exceptional higher occurrence in relapsed/refractory NKTCL patients, indicating potential avenues for targeted combination therapies in these high-risk patients. For example, while HDAC inhibitors have shown efficacy in hematologic malignancies, they often encounter rapid drug resistance [68]. Previous studies have shown that combining HDAC and FGFR inhibition can restore sensitivity to HDAC inhibitors, resulting in synergistic anti-tumor effects [69, 70]. Therefore, for patients with *HDAC9*-*FGFR1* co-occurring mutations, adopting this combined targeted treatment strategy likely provides a highly effective therapeutic option.

Finally, we classified five genetic NKTCL clusters (i.e., C0–C4) with distinct prognosis by integrating potential driver mutations, CNVs, and SVs, which gives a more complete picture on the mutational landscape of NKTCL and better reflects its inherent genetic heterogeneity among patients. The clear distinction among our defined molecular subtypes (i.e., C0–C4) in mutational landscape and prognosis performance calls for novel treatments that target those corresponding driver mutations. Patients in the C0 and C1 clusters exhibited superior clinical outcomes, suggesting them as the best patient group for receiving asparaginase-based chemotherapies that are currently widely used. Mutations involving the JAK-STAT pathway are predominant in the C2 cluster, indicating the patients in this cluster are likely to benefit from treatment with JAK/STAT inhibitors. The C3 cluster is represented by the mutations in epigenetic modifiers, suggesting targeting epigenetic regulators could potentially improve the survival of these patients. Finally, the C4 cluster is characterized by elevated genome instability and high TMB, suggesting patients from this group is likely to benefit from the treatment of genome instability targeted therapy, potentially better in combination with immunotherapy.

## Conclusions

In summary, we comprehensively investigated the genomic characteristics of NKTCL with an emphasis on the comparison between the primary and relapsed/refractory patients. Elevated level of genomic instability was identified for the relapsed/refractory patient group, as reflected by both nucleotide and structural level alterations, including complex events such as chromothripsis, focal amplifications, and eccDNAs. By integrating the recurrent genomic alteration events of full spectrum, we classified NKTCL patients into five groups (C0–C4) with distinct molecular characteristics and clinical prognoses. Taken together, our genomic characterization and molecular subtyping suggest genome instability targeted therapy and better in combination with immune checkpoint therapy will likely provide a promising opportunity for relapsed/refractory NKTCL patients.

### Supplementary Information

**Additional file 1. **Supplementary materials and methods.

**Additional file 2: Table S1.** Summary of the sequencing coverage of the 163 NKTCL tumor samples. **Table S2.** Distribution of clinical features across different patient cohorts (*n* = 163). **Table S3.** Prognostic factors for overall survival (OS) by univariate and multivariate analysis. **Table S4.** The clinical feature summary for the 163 NKTCL patients. **Table S5.** Cancer-related genes interrupted by structural variant (SV) breakpoints in NKTCL patients with WGS data.

**Additional file 3: Fig. S1.** Kaplan–Meier survival curves of overall survival in patients with NKTCL. **Fig. S2.** Characterization of somatic mutations, tumor mutation burden, and MSI status in NKTCL. **Fig. S3.** Mutational landscape of NKTCL. **Fig. S4.** The landscape of copy number variation in NKTCL. **Fig. S5.** The Copy number (CN) signatures identified for NKTCL patients. **Fig. S6.** Sankey-diagram of the molecular subtypes and clinical prognostic models for NKTCL patients.

## Data Availability

The newly generated WES data reported in this paper have been deposited in the Genome Sequence Archive with the accession number of HRA004366 (https://ngdc.cncb.ac.cn/gsa-human/browse/HRA004366) [71]. The publicly available WGS/WES data can be retrieved from European Genome-phenome Archive (EGA) repository with accession number of EGAD00001004140 (https://ega-archive.org/datasets/EGAD00001004140) [20] and EGAS00001002398 (https://ega-archive.org/studies/EGAS00001002398) [19] as well as from The National Omics Data Encyclopedia (NODE) with the accession number of OEP000498 (https://www.biosino.org/node/project/detail/OEP000498) [8]. Custom auxiliary scripts used for results visualization are publicly available at Zenodo (https://doi.org/10.5281/zenodo.10842061) [72].

## Abbreviations

CNVs: Copy number variants
DSBs: Double strand breaks
EBV: Epstein-Barr virus
eccDNA: Extracellular circular DNA
HRD: Homologous recombination deficiency
INDELs: Insertion/deletions
MSI: Microsatellite instability
NHEJ: Non-homologous DNA end-joining
NKTCL: Natural killer/T cell lymphoma
NMF: Non-negative matrix factorization
OS: Overall survival
PD-1: Programmed cell death protein 1
PFS: Progression-free survival
SBS: Single base substitution
SNVs: Single nucleotide variants
SVs: Structural variants
TMB: Tumor mutation burden
WES: Whole-exome sequencing
WGS: Whole-genome sequencing
