# Supplementary material for: Full-spectral genome analysis of natural killer/T cell lymphoma highlights impacts of genome instability in driving its progression
Source: Genome Med. 2024 Apr 2;16:48. doi: 10.1186/s13073-024-01324-5 (PMC10986005; doi:10.1186/s13073-024-01324-5)
Supplement: Supplementary file 1 — Additional file 1. Supplementary materials and methods. [file 13073_2024_1324_MOESM1_ESM.docx]

**Patients and NGS**

Tumor DNA was extracted from formalin-fixed paraffin-embedded (FFPE) tissues from tumor samples using the QIAamp DNA FFPE Tissue Kit (QIAGEN Cat: 56404), while normal DNA was extracted from the paired whole blood samples using the Circulating Nucleic Acid Kit (QIAGEN Cat: 55114). Sequencing libraries were prepared with the Agilent SureSelect XT Human All Exon V5 kit (Santa Clara, CA, USA; Cat: 5190-6209) and KAPA HypePlus kit (KAPA Cat: kk8504). The tumor and normal DNA samples were used for paired-end WES using Illumina HiSeq 4000 (service provided by Geneseeq Technology Inc., Nanjing, China).

**Reference genome and quality control**

We downloaded the human reference genome (vGRCh38) from <ftp://ftp.ncbi.nlm.nih.gov/genomes/all/GCA/000/001/405/GCA_000001405.15_GRCh38/seqs_for_alignment_pipelines.ucsc_ids/GCA_000001405.15_GRCh38_no_alt_analysis_set.fna.gz> . Careful quality control was applied during our data integration and processing. Briefly, starting with the raw reads, we applied adapter and quality trimming with Trimmomatic (version 0.38)^1^ and only used the high-quality part of the reads for read mapping. All WGS and WES reads were mapped to the human reference genome (vGRCh38) with bwa (v0.7.17)^2^. The resulting BAM file was further processed with samtools (v1.14)^3^, Picard tools (v2.26.4, https://broadinstitute.github.io/picard/), and GATK3 (v3.6-6)^4^ for sorting, indexing, and local realignment. A minimal mapping quality score of 30 was applied to keep only reads that were highly confidently aligned.

**Somatic SNV and INDEL calling**

Somatic SNVs and INDELs were detected with MuTect2 (v4.1.0.0)^5^ with a variant quality score cutoff of 20 and annotated by ANNOVAR (v2020Jun08)^6^. To filter the potential germline variants, we retrieved the common germline variants (allele frequency>1%) identified in the 1,000 Genomes project (http://www.openbioinformatics.org/annovar/download/hg38_1000g2015aug.zip), the Exome Aggregation Consortium (https://www.openbioinformatics.org/annovar/download/hg38_exac03.txt.gz), and the Genome Aggregation Database (https://www.openbioinformatics.org/annovar/download/hg38_gnomad_genome.txt.gz) and filtered out the matched somatic variants. In addition, we also created an in-house normal control variant database by taking called variants in at least two normal control samples used in this study. Called somatic variants from tumor samples that matched with these variants were further discarded. We further removed somatic variants with an alternate allele depth (AD) <3 in the WGS data and an alternate AD <5 in the WES data, or variant allele frequency (VAF) <5%. MAFtools (v2.14.0)^7^ and the ComplexHeatmap (v2.14.0)^8^ were used for visualization and tumor mutational burden (TMB) estimation.

The mutational landscape is categorized based on molecular functions into genome instability associated genes, epigenetic modifiers, JAK/STAT signaling, RNA helicase, and tumor suppressors. We defined genome instability associated genes by incorporating genes associated with DNA repair, mitotic checkpoint, and mitotic spindle in the MSigDB hallmark gene set^9^ as well as genes linked to genome instability in the harmonizome dataset^10^. The Epigenetic modifiers were defined based on the database of epigenetic modifiers (dbEM)^11^. The JAK/STAT signaling gene set was defined based on genes associated with this signaling pathway in the MSigDB hallmark gene set^9^. The RNA helicase gene set is defined based on genes associated with RNA helicase activity in their Gene Ontology (GO) Annotations^12, 13^. Finally, the tumor suppressors were defined based on the database for tumor suppressor genes (TSGene 2.0)^14^.

Single base substitution (SBS) signatures and small insertions and deletions (ID) signatures were extracted and visualized by SigProfile (v1.2.14)^15^ based on Catalogue of Somatic Mutations in Cancer (COSMIC)^16^.

**CNVs and SVs calling**

Somatic CNVs were primarily called using CNVkit (v0.9.9)^17^. Allele-specific CNVs was further evaluated by ASCAT (v3.1.1)^18^ and used for Copy number (CN) signature extraction by SigProfile (v1.2.14)^15^. The KEGG pathway (https://www.genome.jp/kegg/pathway.html) enrichment analysis for gene-level CNVs was performed by clusterProfiler (v4.7.1.3)^19^. The aneuploidy score was calculated using the get_Aneuploidy_score() function implemented in sigminer (v2.1.9)^20^.

For WGS data, canonical SVs were detected using manta (v1.6.0)^21^ following the instruction of the software. Furthermore, we performed cancer-related gene annotation for genes that were disrupted by SV breakpoints based on the MSigDB hallmark gene sets^9^ relevant to cancer-related processes. Such processes include angiogenesis, apoptosis, DNA repair, E2F targets, epithelial mesenchymal transition, G2M checkpoint, hedgehog signaling, IL2-STAT5 signaling, IL6-JAK-STAT3 signaling, KRAS signaling, mitotic spindle, MTORC1 signaling, NOTCH signaling, P53 pathway, PI3K-AKT-MTOR signaling, TGF beta signaling, TNFA signaling via NFKB, and WNT-beta catenin signaling.

For complex SVs, we examined chromothripsis events with ShatterSeek (v1.1)^22^ based on the afore-identified CNVs and canonical SVs results. High-confidence candidates were further defined as having >=6 interleaved SVs and >=7 contiguous segments oscillating between two copy number states. For focal amplifications, we used AmpliconArchitect (v1.3.r1)^23^ (criteria: CN>=5 and size>=100 kb) and AmpliconClassifier (v0.4.13)^24^ for our analysis, with the former for event detection and the latter for event classification: circular (i.e., eccDNA), breakage-fusion-bridge (BFB), linear, or complex.

1. Bolger AM, Lohse M, Usadel B. Trimmomatic: a flexible trimmer for Illumina sequence data. *Bioinformatics (Oxford, England)* 2014 Aug 1; **30**(15)**:** 2114-2120.

2. Li H, Durbin R. Fast and accurate short read alignment with Burrows-Wheeler transform. *Bioinformatics (Oxford, England)* 2009 Jul 15; **25**(14)**:** 1754-1760.

3. Danecek P, Bonfield JK, Liddle J, Marshall J, Ohan V, Pollard MO*, et al.* Twelve years of SAMtools and BCFtools. *GigaScience* 2021 Feb 16; **10**(2).

4. Van der Auwera GA, Carneiro MO, Hartl C, Poplin R, Del Angel G, Levy-Moonshine A*, et al.* From FastQ data to high confidence variant calls: the Genome Analysis Toolkit best practices pipeline. *Current protocols in bioinformatics* 2013; **43**(1110)**:** 11.10.11-11.10.33.

5. Cibulskis K, Lawrence MS, Carter SL, Sivachenko A, Jaffe D, Sougnez C*, et al.* Sensitive detection of somatic point mutations in impure and heterogeneous cancer samples. *Nature biotechnology* 2013 Mar; **31**(3)**:** 213-219.

6. Wang K, Li M, Hakonarson H. ANNOVAR: functional annotation of genetic variants from high-throughput sequencing data. *Nucleic acids research* 2010 Sep; **38**(16)**:** e164.

7. Mayakonda A, Lin DC, Assenov Y, Plass C, Koeffler HP. Maftools: efficient and comprehensive analysis of somatic variants in cancer. *Genome research* 2018 Nov; **28**(11)**:** 1747-1756.

8. Gu Z, Eils R, Schlesner M. Complex heatmaps reveal patterns and correlations in multidimensional genomic data. *Bioinformatics (Oxford, England)* 2016 Sep 15; **32**(18)**:** 2847-2849.

9. Liberzon A, Birger C, Thorvaldsdóttir H, Ghandi M, Mesirov JP, Tamayo P. The Molecular Signatures Database (MSigDB) hallmark gene set collection. *Cell systems* 2015 Dec 23; **1**(6)**:** 417-425.

10. Rouillard AD, Gundersen GW, Fernandez NF, Wang Z, Monteiro CD, McDermott MG*, et al.* The harmonizome: a collection of processed datasets gathered to serve and mine knowledge about genes and proteins. *Database : the journal of biological databases and curation* 2016; **2016**.

11. Singh Nanda J, Kumar R, Raghava GP. dbEM: A database of epigenetic modifiers curated from cancerous and normal genomes. *Scientific reports* 2016 Jan 18; **6:** 19340.

12. Ashburner M, Ball CA, Blake JA, Botstein D, Butler H, Cherry JM*, et al.* Gene ontology: tool for the unification of biology. The Gene Ontology Consortium. *Nature genetics* 2000 May; **25**(1)**:** 25-29.

13. Aleksander SA, Balhoff J, Carbon S, Cherry JM, Drabkin HJ, Ebert D*, et al.* The Gene Ontology knowledgebase in 2023. *Genetics* 2023 May 4; **224**(1).

14. Zhao M, Kim P, Mitra R, Zhao J, Zhao Z. TSGene 2.0: an updated literature-based knowledgebase for tumor suppressor genes. *Nucleic acids research* 2016 Jan 4; **44**(D1)**:** D1023-1031.

15. Bergstrom EN, Huang MN, Mahto U, Barnes M, Stratton MR, Rozen SG*, et al.* SigProfilerMatrixGenerator: a tool for visualizing and exploring patterns of small mutational events. *BMC genomics* 2019 Aug 30; **20**(1)**:** 685.

16. Alexandrov LB, Kim J, Haradhvala NJ, Huang MN, Tian Ng AW, Wu Y*, et al.* The repertoire of mutational signatures in human cancer. *Nature* 2020 Feb; **578**(7793)**:** 94-101.

17. Talevich E, Shain AH, Botton T, Bastian BC. CNVkit: Genome-Wide Copy Number Detection and Visualization from Targeted DNA Sequencing. *PLoS computational biology* 2016 Apr; **12**(4)**:** e1004873.

18. Van Loo P, Nordgard SH, Lingjærde OC, Russnes HG, Rye IH, Sun W*, et al.* Allele-specific copy number analysis of tumors. *Proceedings of the National Academy of Sciences of the United States of America* 2010 Sep 28; **107**(39)**:** 16910-16915.

19. Yu G, Wang LG, Han Y, He QY. clusterProfiler: an R package for comparing biological themes among gene clusters. *Omics : a journal of integrative biology* 2012 May; **16**(5)**:** 284-287.

20. Wang S, Li H, Song M, Tao Z, Wu T, He Z*, et al.* Copy number signature analysis tool and its application in prostate cancer reveals distinct mutational processes and clinical outcomes. *PLoS genetics* 2021 May; **17**(5)**:** e1009557.

21. Chen X, Schulz-Trieglaff O, Shaw R, Barnes B, Schlesinger F, Källberg M*, et al.* Manta: rapid detection of structural variants and indels for germline and cancer sequencing applications. *Bioinformatics (Oxford, England)* 2016 Apr 15; **32**(8)**:** 1220-1222.

22. Cortés-Ciriano I, Lee JJ, Xi R, Jain D, Jung YL, Yang L*, et al.* Comprehensive analysis of chromothripsis in 2,658 human cancers using whole-genome sequencing. *Nature genetics* 2020 Mar; **52**(3)**:** 331-341.

23. Deshpande V, Luebeck J, Nguyen ND, Bakhtiari M, Turner KM, Schwab R*, et al.* Exploring the landscape of focal amplifications in cancer using AmpliconArchitect. *Nature communications* 2019 Jan 23; **10**(1)**:** 392.

24. Luebeck J, Ng AWT, Galipeau PC, Li X, Sanchez CA, Katz-Summercorn AC*, et al.* Extrachromosomal DNA in the cancerous transformation of Barrett's oesophagus. *Nature* 2023 Apr 12.
