# Supplementary material for: Full-spectral genome analysis of natural killer/T cell lymphoma highlights impacts of genome instability in driving its progression
Source: Genome Med. 2024 Apr 2;16:48. doi: 10.1186/s13073-024-01324-5 (PMC10986005; doi:10.1186/s13073-024-01324-5)
Supplement: Supplementary file 3 — Additional file 3: Fig. S1. Kaplan–Meier survival curves of overall survival in patients with NKTCL. Fig. S2. Characterization of somatic mutations, tumor mutation burden, and MSI status in NKTCL. Fig. S3. Mutational landscape of NKTCL. Fig. S4. The landscape of copy number variation in NKTCL. Fig. S5. The Copy number (CN) signatures identified for NKTCL patients. Fig. S6. Sankey-diagram of the molecular subtypes and clinical prognostic models for NKTCL patients. [file 13073_2024_1324_MOESM3_ESM.docx]

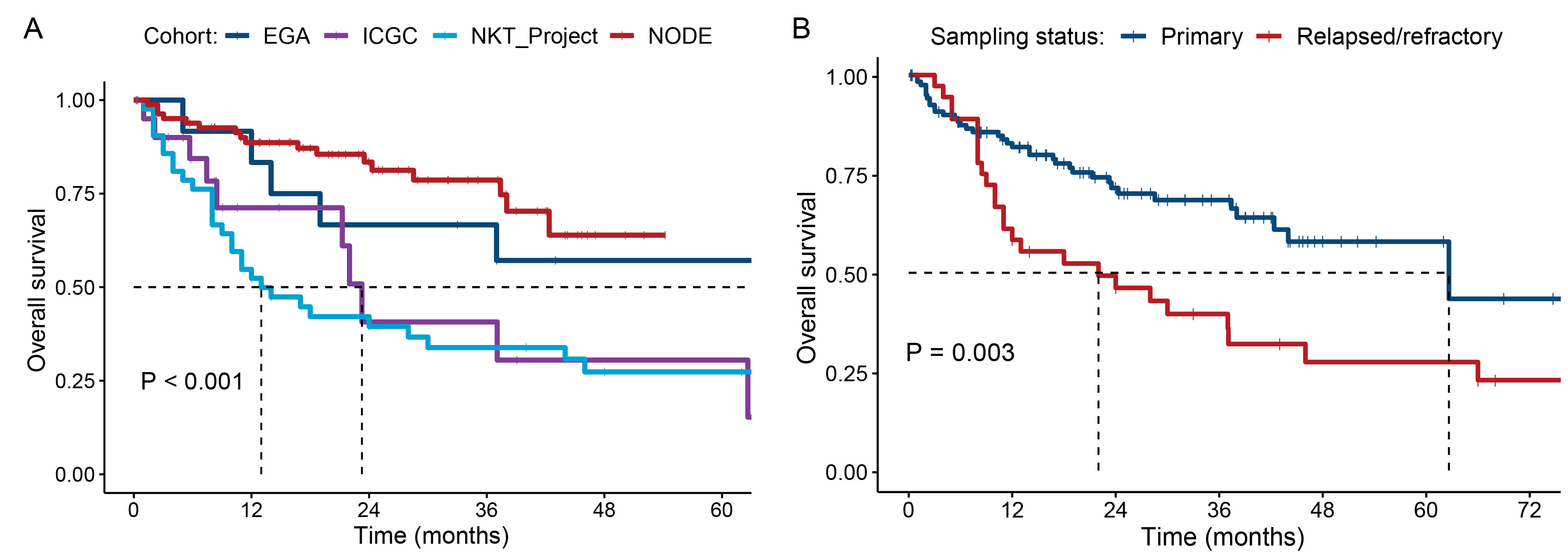


**Fig. S1. Kaplan–Meier survival curves of overall survival in patients with NKTCL.** (A) Stratified by different patient cohorts. (B) Stratified by sampling status (primary vs. relapsed/refractory).


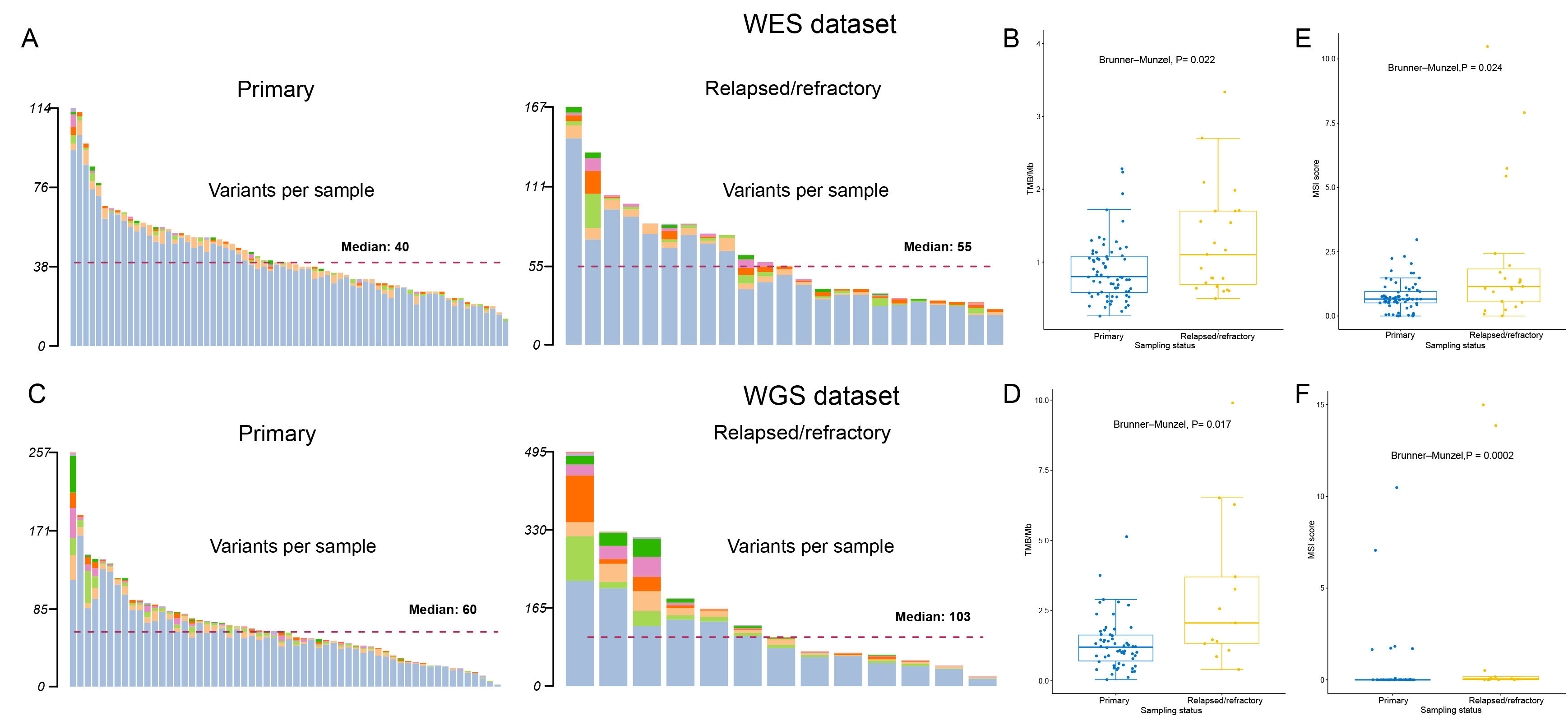


**Fig. S2. Characterization of somatic mutations, tumor mutation burden, and MSI status in NKTCL.** Per-sample number distribution of different categories of non-synonymous mutations in patients with primary (left) and relapsed/refractory (right) NKTCL in the WES dataset (A) and WGS dataset (C). The dashed line denotes the median value. The comparison of TMB levels between the primary and the relapsed/refractory group in the WES dataset (B) and WGS dataset (D). The comparison of MSI score between the primary and the relapsed/refractory group in the WES dataset (E) and WGS dataset (F). Brunner–Munzel test was applied for assessing the statistical significance.


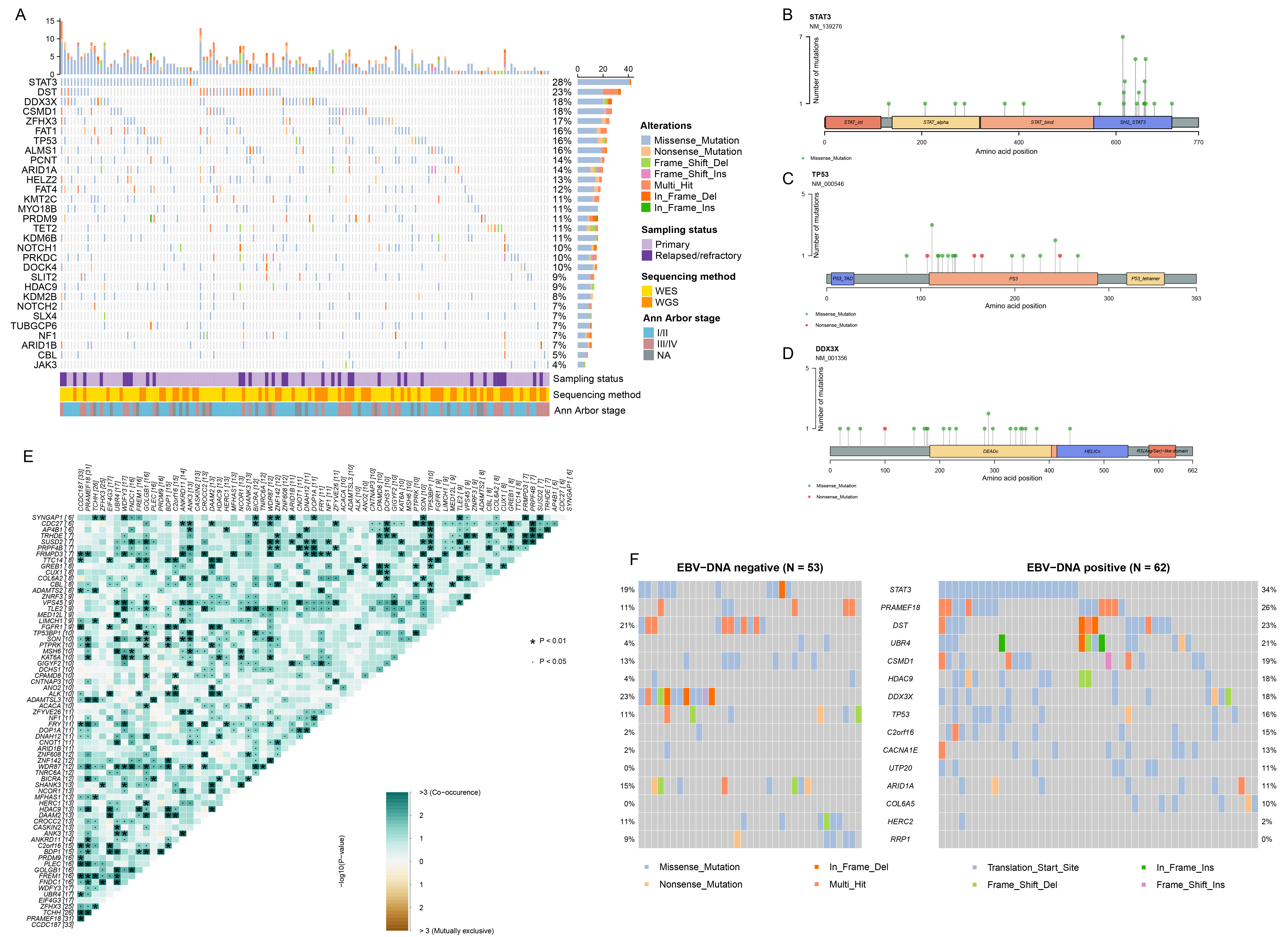


**Fig. S3. Mutational landscape of NKTCL.** (A) The mutational landscape of frequent somatic mutations in the 163 NKTCL patients. The mutated genes are ranked by the mutational frequency. **The** lollipop plot of the somatic mutations occurred in *STAT3* (B), *TP53* (C), and  *DDX3X*(D). (E) The co-occurrence and mutual exclusivity patterns among the 68 genes more likely to be mutated in relapsed/refractory patients. (F) The comparison of mutational landscape of EBV-DNA negative patients and EBV-DNA positive patients. The mutational frequency of these genes in EBV-DNA negative patients are shown on the left, while their corresponding mutational frequencies in EBV-DNA positive patients are shown on the right. The mutated genes are ranked by the mutational frequency in the latter group.


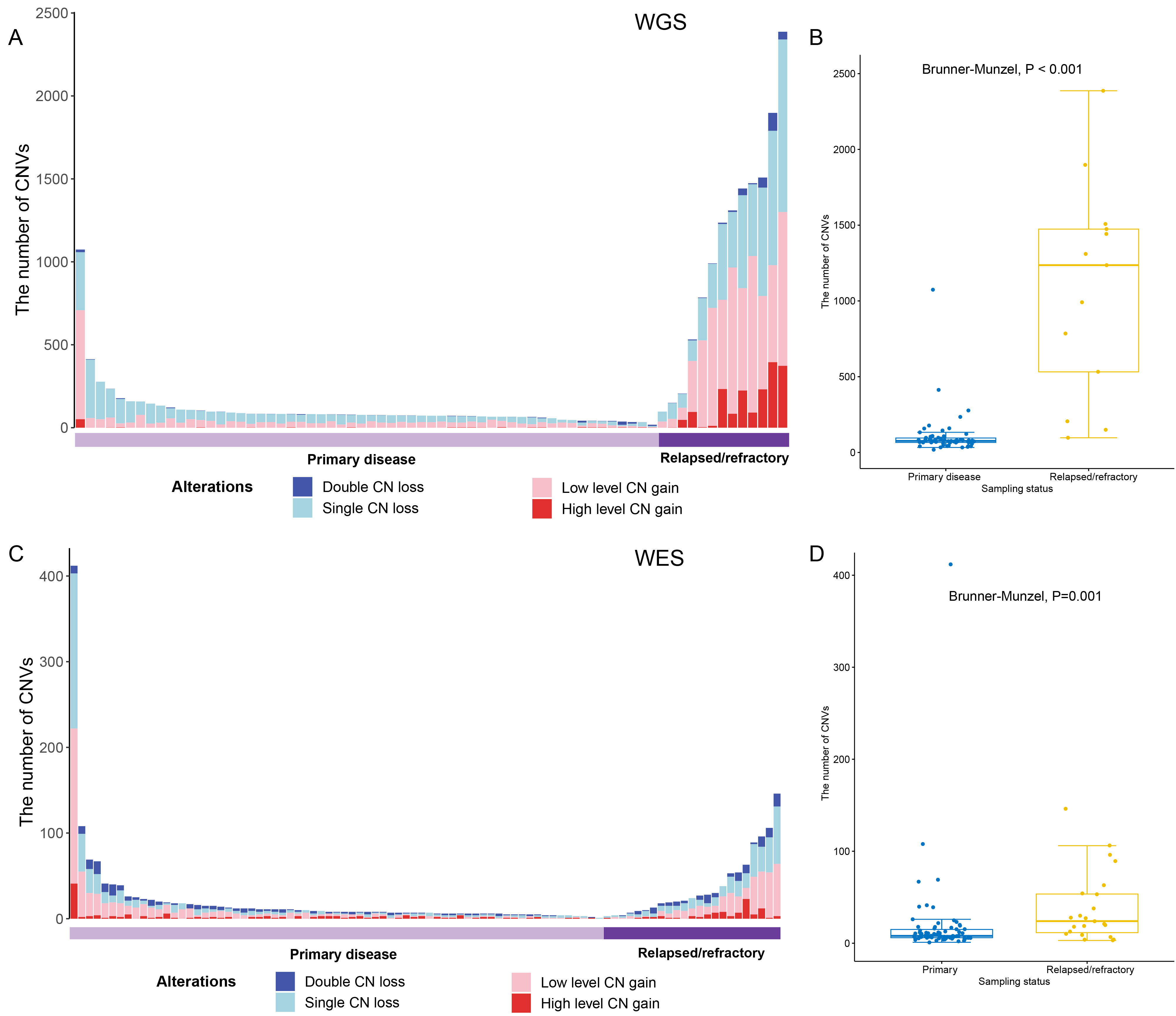


**Fig. S4. The landscape of copy number variation in NKTCL**. Distribution of different categories of CNVs across the 71 NKTCL patients with whole-genome sequencing (WGS) data (A), and 92 patients with whole-exome sequencing (WES) data (C). The CNV categories employed here include single CN loss (CN=1 copy), double CN loss (CN=0), low-level CN gain (CN=3) and high-level CN gain (CN>3 copies). The comparison of per-sample CNVs numbers between the primary and relapsed/refractory patient in WGS groups (B) and WES groups (D). Brunner–Munzel test was applied for assessing the statistical significance.


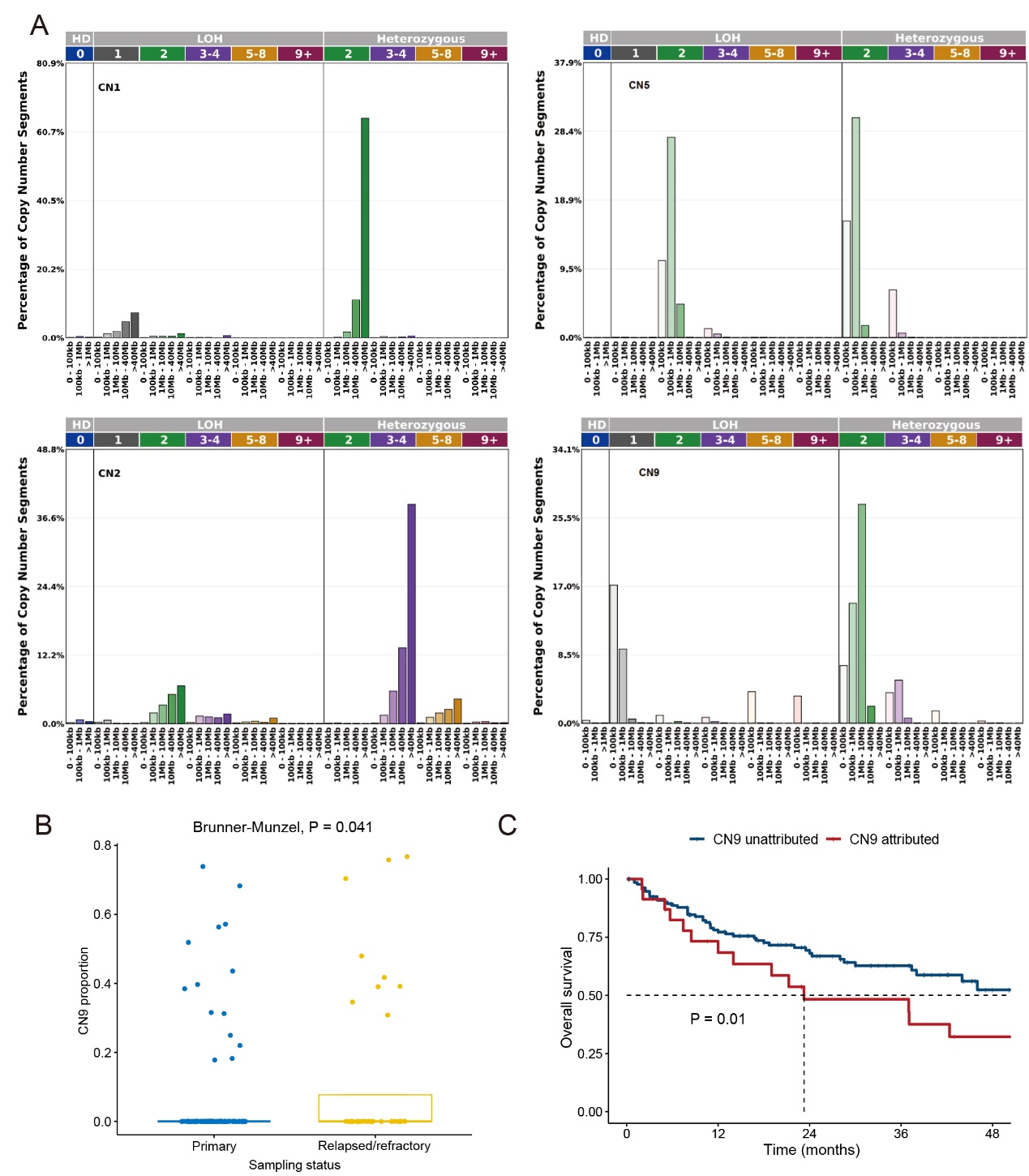


**Fig. S5. The Copy number (CN) signatures identified for NKTCL patients.** (A) Decomposition plots of prominent CN signatures (CN1, CN2, CN5 and CN9). The exact copy number (0–9+) and heterozygosity status (HD: homozygously deleted, LOH: loss of heterozygosity, Heterozygous: heterozygously deleted) are shown on the top, while the corresponding segment sizes are denoted at the bottom. (B) The proportion of CNVs with the CN9 signature between the primary and relapsed/refractory NKTCL patients. The relapsed/refractory group significantly detects more CN9 than the primary group (P = 0.041, Brunner-Munzel test). (C) Kaplan–Meier curves of overall survival (OS) by the attribute of CN9 in all examined NKTCL patients.


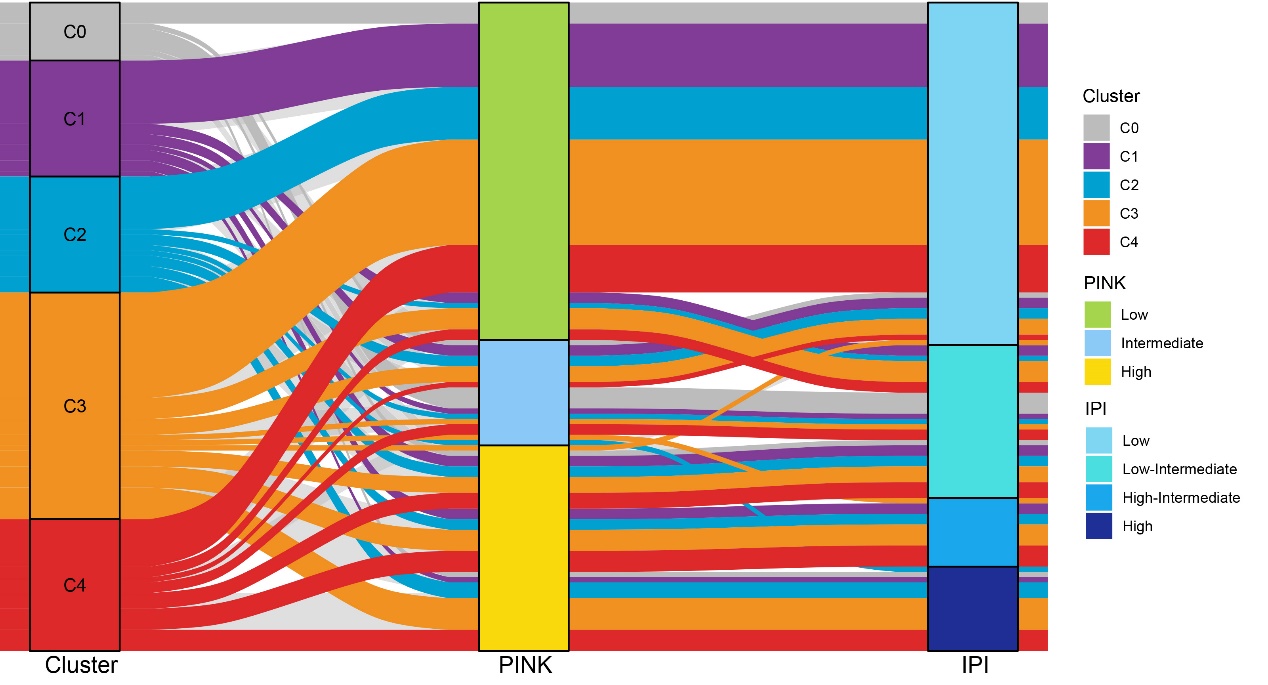


**Fig. S6. Sankey-diagram of the molecular subtypes and clinical prognostic models for NKTCL patients.** The three columns from the left to the right represent molecular subtyping clusters, PINK scores, and IPI scores respectively, with the total height represents the 123 NKTCL patient samples with retrievable IPI and PINK scores. The curves with different colors show the correspondence relationship among different molecular subtyping clusters and the PINK and IPI prognostic models.
